# Garden of Eden

**Published:** 1870-10

**Authors:** 


### GARDEN OF EDEN.

At the junction of the Euphrates and Tigris rivers is a mean little Arab village called Korneh, containing about two hundred houses stretched along the river bank, made of canes and covered with straw. At the extreme point, just where the rivers meet, is a telegraphic station. Hence the general supposed location of Eden is associated with the telegraph of Morse, whom we may well suppose would have missed arriving at the Heavenly Eden had he yielded to suicide, which he once strongly contemplated on an occasion when he found himself so beset with difficulties and discouragements that life had lost all its attractions ; but some good angel snatched him away from the dreadful precipice.

